# Visualization of Annular Gap Junction Vesicle Processing: The Interplay Between Annular Gap Junctions and Mitochondria

**DOI:** 10.3390/ijms20010044

**Published:** 2018-12-22

**Authors:** Cheryl L. Bell, Teresa I. Shakespeare, Amber R. Smith, Sandra A. Murray

**Affiliations:** 1 Department of Cell Biology, School of Medicine, University of Pittsburgh, Pittsburgh, PA 15261, USA; clb206@pitt.edu (C.L.B.); ambersmith@pitt.edu (A.R.S.); 2 Department of Biology, Savannah State University, Savannah, GA 31404, USA; teresa.shakespeare@gmail.com

**Keywords:** gap junction, connexin, annular gap junction vesicle, mitochondria, lysosome

## Abstract

It is becoming clear that in addition to gap junctions playing a role in cell–cell communication, gap junction proteins (connexins) located in cytoplasmic compartments may have other important functions. Mitochondrial connexin 43 (Cx43) is increased after ischemic preconditioning and has been suggested to play a protective role in the heart. How Cx43 traffics to the mitochondria and the interactions of mitochondria with other Cx43-containing structures are unclear. In this study, immunocytochemical, super-resolution, and transmission electron microscopy were used to detect cytoplasmic Cx43-containing structures and to demonstrate their interactions with other cytoplasmic organelles. The most prominent cytoplasmic Cx43-containing structures—annular gap junctions—were demonstrated to form intimate associations with lysosomes as well as with mitochondria. Surprisingly, the frequency of associations between mitochondria and annular gap junctions was greater than that between lysosomes and annular gap junctions. The benefits of annular gap junction/mitochondrial associations are not known. However, it is tempting to suggest, among other possibilities, that the contact between annular gap junction vesicles and mitochondria facilitates Cx43 delivery to the mitochondria. Furthermore, it points to the need for investigating annular gap junctions as more than only vesicles destined for degradation.

## 1. Introduction

Gap junction channels play a pivotal role in a vast number of physiological events by providing channels for the intercellular communication of regulatory molecules between cells [1]. Gap junction channels are composed of gap junction proteins, termed connexins [2]. All of the members of the multigene connexin family have a similar topology and the sequences of several connexin gap junction proteins have been determined [3,4]. Twenty-one different connexins have been identified in humans [5]. Connexin 43 (Cx43) gap junction protein, the most ubiquitously expressed connexin, is thought to be synthesized in the endoplasmic reticulum, oligomerized into a hemichannel in the Golgi [6] and then transported to the cell surface in secretory vesicles. On the cell surface, hemichannels from apposing cells align (dock) to form channels [7]. Gap junction channels then aggregate to form gap junction plaques [1]. These gap junction plaques are composed of thousands of channels [6] and plaque size is determined by the number of hemichannels that are delivered to the membrane in secretory vesicles and aggregated on the surface to form these plaques [7].

Gap junction plaques are removed from the cell surface by an internalization event [8,9]. The disassembly and removal of gap junction plaques from the plasma membrane involve a clathrin/dynamin-dependent unique internalization process [10,11,12] in which the gap junction plaque that connects two adjacent cells is internalized into the cytoplasm of one of the two cells to form an annular gap junction [13,14,15,16]. These annular gap junctions were first identified with transmission electron microscopy (TEM) and were distinguished from other organelles by the presence of a double-membrane (pentalaminar membrane) and a central lumen [8,13,14,15,16,17,18]. Annular gap junctions (also called connexosomes) have now been demonstrated with live cell imaging techniques to form from internalization at central regions of the gap junction plaque [19,20,21,22] or internalization of the entire gap junction plaque [23].

The central dogma has been that the gap junction proteins (connexins) in these annular gap junctions are degraded by autophagosomal and endo/lysosomal pathways [24,25,26,27,28,29], and thus the internalization process is only a method of eliminating old connexins [8,15,30]. However, data from our laboratory and that of others has demonstrated that connexins, specifically annular gap junction vesicle connexin 43 (Cx43), can recycle back to the cell surface to form functional gap junctions [31,32]. While the aggregation of gap junction channels into plaques and channel gating have been extensively studied [19,27,28], mechanisms involved in the processing of annular gap junction vesicles, and the organelles they interact with, have only recently gained attention. However, it is clear that the rates of gap junction plaque disassembly as well as organelle interactions are critical to gap junction function in cell–cell communication and cell–cell adhesion [29,30]. Further, changes in gap junction location, connexin trafficking, and internalization is thought to play several pivotal physiological roles during embryonic development [33,34], mitosis [32], wound healing [35,36], and cell migration [35]. Furthermore, gap junction plaque changes are thought to facilitate tumor growth and metastasis [34] as well as play a pivotal role in the cardiac electrophysiology and the survival of cardiomyocyte during ischemia [37]. Additionally, Cx43, located in mitochondria, is thought to regulate mitochondrial function (oxygen consumption and potassium fluxes) [38]. Mitochondrial Cx43 is altered under several pathophysiological cardiac conditions including, hypertension, hypertrophy, hypercholesterolemia, ischemia/reperfusion injury, post-infarction remodeling, and heart failure [38,39,40,41,42]. The possible pathophysiological role of Cx43 within cytoplasmic compartments or associations between these compartments with mitochondria is not known. Understanding Cx43 trafficking, fate and the translocation to Cx43-containing organelles is essential to an understanding of a host of normal and pathological events.

In this study, we demonstrated cytoplasmic Cx43-containing structures and evaluated their organelle interactions. We found that the most abundant Cx43-containing structures, annular gap junction vesicles, associated more frequently with mitochondria than with lysosomes. These findings are consistent with the possibility that annular gap junction and mitochondria may communicate with one another and that annular gap junction Cx43 may be utilized rather than being only degraded.

## 2. Results

### 2.1. Characterization of Cx43-Containing Gap Junctions’ Structure Behavior

**Figure 1:**
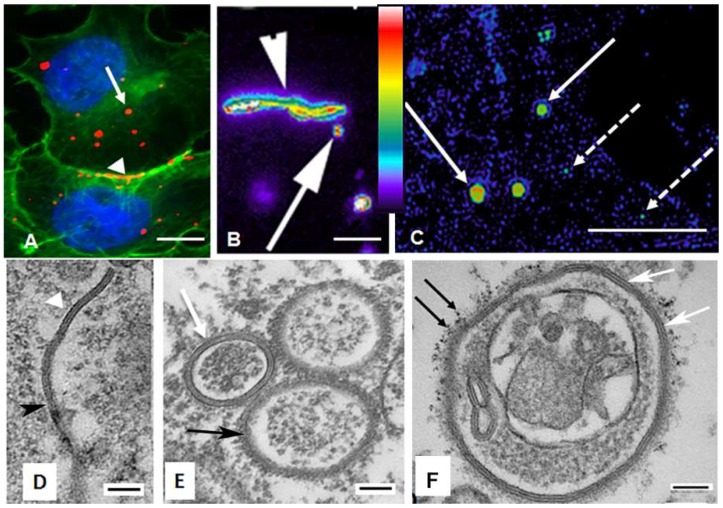
Localization of gap junction plaques and annular gap junction vesicles. (**A**) Immunocytochemistry of endogenous Cx43 (red) and cortical actin (green). Cortical actin (green) staining was used to define the boundaries of the cell and to aid in distinguishing intracellular annular gap junction puncta (red, arrow) from surface gap junction plaques (red, arrowhead). (**B**) Pseudocolored confocal microscopic image of Cx43-GFP from a Time-lapse frame with gap junction plaques (arrowhead) and annular gap junction vesicles (arrow) demonstrate the differences in Cx43-GFP intensities. (**C**) Pseudocolored super-resolution microscopic image demonstrating Cx43-GFP intensities of annular gap junction (arrow) and secretory vesicles (broken arrow). The Look Up Table (LUT) scale in B and C indicates the intensities of GFP in the gap junction structures. (**D**,**E**) TEM of a gap junction plaque (**D**) and annular gap junction vesicles (**E**) identified, depending on the plane of focus, by typical pentalaminar membrane (white arrow head or arrow) or the appearance of the densely stained double membrane (black arrowhead and arrow). (**F**) Bizarre annular gap junction vesicle with annular structure and other unknown material within the lumen. The pentalaminar membrane (white arrows) and immuno-electron microscopic Cx-43 Q-dots labeling (black arrows) was used to identify the annular gap junction vesicle. Scale Bars = (**A**,**B**):10 µm, (**C**): 5 µm, (**D**–**F**): 100 nm.

**Figure 2:**
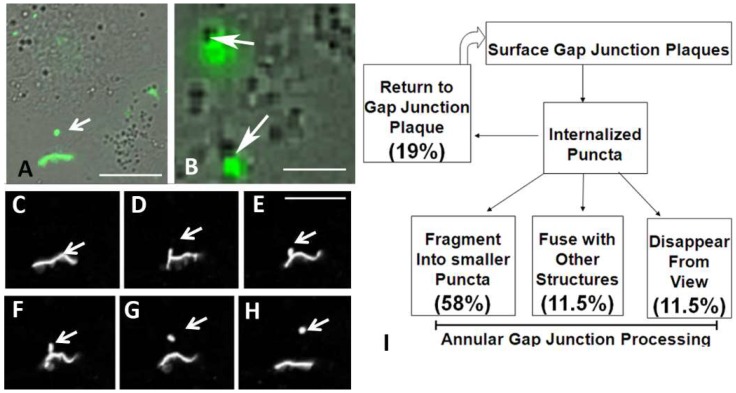
Time-lapse imaging of transfected cells expressing Cx43-GFP. (**A**,**B**) Differential interference contrast (DIC)/fluorescein isothiocyanate (FITC) overlay images of cell populations expressing Cx43-GFP. (**A**) An annular gap junction vesicle (arrow) and gap junction plaque (green) are evident. (**B**) Annular gap junctions (green) that were in contact with unidentified dense cytoplasmic structures (arrows) can be seen. (**C**–**H**) Montage of selected Time-lapse images collected at 1 min intervals. The gap junction plaque between two cells is evident as well as the cytoplasmic annular gap junction (arrows, (**G**,**H**)). The invagination of the gap junction plaque (arrows, (**C**–**F**)) and the subsequent release of an annular gap junction vesicle into the cytoplasm of one of the two contacting cells could be followed over time and the cell morphology appreciated by the DIC/FITC over lay image (**A**). (**I**) Flow chart summary of Time-lapse observations depicting annular gap junction formation and behavior. Scale Bars (**A**–**H**): 10 µm.

A human adrenal tumor cell line (SW-13) that expresses Cx43 gap junction protein was used throughout this study to analyze cytoplasmic Cx43-containing structures, particularly that of annular gap junction vesicles, which are the most prominent Cx43-containing organelle in the cytoplasm (Figure 1A). This cell line forms large gap junctions that are spontaneously internalized to form annular gap junction vesicles, which is particularly advantageous for studying aspects of Cx43 trafficking. With immunocytochemical labeling of cells expressing endogenous Cx43 and live cell imaging of cells expressing Cx43-GFP, gap junction plaques can be seen at the cell surface between contacting cells (Figure 1A,B and Figure 2). Puncta indicative of annular gap junction vesicles could be easily seen within the cytoplasm (Figure 1 and Figure 2). In addition, secretory vesicles that deliver new Cx43 to the plasma membrane could be detected at the light level of resolution, but they were more easily discerned with super-resolution microscopy (Figure 1C). Secretory and annular gap junction vesicles both appeared as puncta at the light microscopic level of resolution and are distinguished from one another mainly by the differences in their sizes. Annular gap junction vesicles (≥0.5 µm) have been previously demonstrated to be much larger than secretory vesicles (≤150 nm) [19,40].

With transmission electron microscopy gap junction plaques as well as annular gap junction vesicles could be positively identified, depending on the plane of focus, by either the typical pentalaminar membrane (Figure 1D,E, white arrow or arrow head) or the densely stained appearance of the double membrane (Figure 1D,E, black arrow or arrow head).

In addition to annular gap junctions and secretory vesicles, ultrastructural analysis revealed atypical aggregates of gap junction membrane vesicles (Figure 1F). These structures were identified by the typical gap junction membrane while the more bizarre structures were further confirmed as composed of Cx43 with Q dot immuno-electron microscopy (Figure 1F). Such structures were very rare with an occurrence of less than 10 in 549 images of annular, 0.02%. Given the recent suggestion that Cx43 in annular gap junctions could have fates other than only degradation [31,32], we did a detailed analysis of annular gap junction processing and their associations with other organelles.

#### Live Cell Imaging of Gap Junction Plaque Endoexocytosis in Cells Expressing Cx43-GFP

Since at the light microscopic level distinguishing the pentalaminar or dense double membranes was not possible, positive identification of annular gap junctions in these live cell imaging studies was made by monitoring gap junction plaque internalization and thus following annular gap junctions observed to form in cells expressing Cx43-GFP. Annular gap junctions were observed to form both as a result of internalization of portions of gap junction plaques or entire gap junction plaques. In both cases the annular gap junction vesicle was released into one of two contacting cells (Figure 2). We were unable to track or analyze the smaller gap junction vesicles since they quickly went out of focus. Of the annular gap junctions that were tracked, the average puncta size, measured from live cell imaging of Cx43-GFP expressing cells, was 1.6 ± 0.3 μm^2^, a size previously shown to be consistent with annular gap junctions and not secretory vesicles [19]. In Figure 2, a gap junction plaque is shown to first invaginate to produce a gap junction bud which in time detaches to form an annular gap junction vesicle. Over a 7 h viewing period 24.0 ± 3.4 annular gap junctions were observed to form.

As summarized in Figure 2H, over a 26 h monitoring period, 93 annular gap junctions, selected for tracking analysis, from four different movies, were observed to either (a) fragment to form smaller puncta, (b) join with cytoplasmic organelles, (c) disappear from view, or (d) move toward and appear to associate with the cell surface gap junction plaques. Note, while other puncta were observed to be released into the cytoplasm and remain relatively stationary, we did not quantitate their number. Since annular gap junctions, observed with FITC fluorescent and DIC imaging, were seen to join with other cytoplasmic organelles (Figure 2B), we were prompted to determine the identity of the structures that associated with the annular gap junction vesicles.

### 2.2. Annular Gap Junction Fusion with Other Organelles

**Figure 3:**
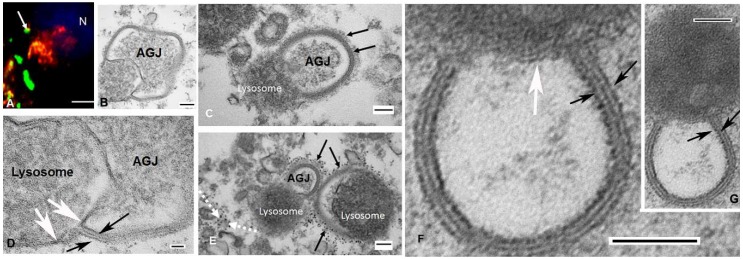
Localization of annular gap junction vesicles and lysosomes. (**A**) Immunocytochemical localization of endogenous Cx43 (green) and lysosomes detected with LAMP1 (red) demonstrates the colocalization (yellow) of an annular gap junction vesicle and lysosome (arrow). (**B**–**G**) The intimate contact between annular gap junction (AGJ) and lysosomes, which are in various stages of degradation is shown. In **B** (and enlarged in **D**) the outer membrane of the annular gap junction can be seen to be continuous with that of the lysosomal membrane (white arrows). Clathrin, identified by the bristle coat (arrows, (**C**)) or quantum dot label (black arrows, (**E**)), can be seen associated with annular gap junction vesicles that are fused with lysosomes. Typical clathrin-coated vesicles also can be seen decorated with clathrin Q dots (dashed white arrows in (**E**)). (**F**) (enlarged from (**G**)) and (**D**) (enlarged from (**B**)) have been magnified to allow the annular gap junction pentalaminar membranes to be clearly seen (black arrows) and the change in this membrane at the annular/lysosomal junction to be appreciated (white arrows in (**F**,**D**)) N = Nucleus; Scale Bars = (**A**): 10 µm, (**B**,**C**,**E**–**G**): 100 nm, (**D**): 20 nm.

**Figure 4:**
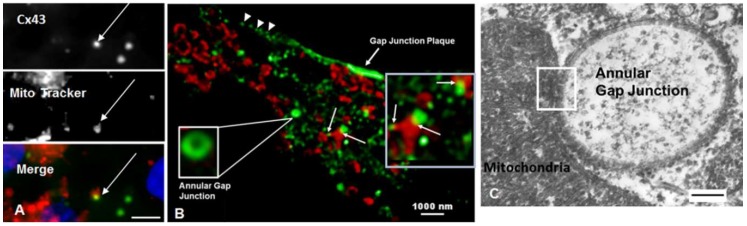
Mitochondria associate with annular gap junctions as seen with standard light (**A**), super-resolution STED (**B**), and TEM microscopy (**C**). (**A**,**B**) Immunocytochemical analysis of mitochondria, with Mitotracker (red in (**A**)) or with antibody to TOM (red in (**B**)) and endogenous Cx43 gap junction antigen detected with antibody staining (green in (**A**,**B**)). The colocalization (yellow, white arrows) of mitochondria (red) and annular gap junctions (green) can be seen. (**B**) Note the gap junction plaque as well as small puncta at the cell surface (arrowheads) that are indicative of gap junction plaque formation. The image in (**B**) has been magnified three times (boxes) to show the lumen of an annular gap junction vesicle and the contact between mitochondria and annular gap junctions yellow, white arrows). (**C**) The intimate association between the mitochondria and annular gap junction (white box) seen with TEM. Scale bars = (**A**): 10 µm, (**B**): 1000 nm, (**C**) 100 nm.

**Figure 5:**
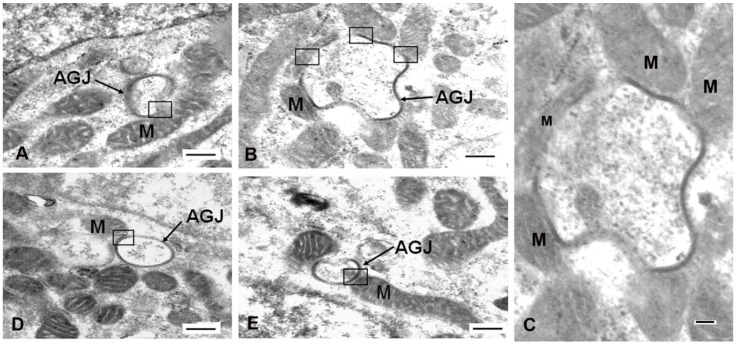
Transmission electron microscopy of mitochondria closely associated with annular gap junctions. (**A**–**E**) Areas of contact between annular gap junctions (AGJ) and mitochondria (M) are indicated within the black boxes (**A**,**B**,**D**,**E**). (**C**) The annular gap junction vesicle seen in (**B**), but imaged at a different section plane, is enlarged to better show the contact areas. Scale Bars: 500 nm.

In cells expressing endogenous Cx43 as well as those transfected to express Cx43-GFP, annular gap junctions were observed, with immunocytochemical colocalization techniques, to associate with two different organelles, lysosomes (Figure 3) as expected, and mitochondria (Figure 4 and Figure 5). The details of these interactions were analyzed.

#### 2.2.1. Analysis of Lysosomes/Annular Gap Junction Associations

Lysosomes, detected with a lysosomal marker, Lamp 1, were shown to colocalize with annular gap junctions in cells expressing endogenous Cx43 (Figure 3A). We counted the total number of connexin puncta and calculated the percentage that was observed to be in contact with lysosomes. Analysis of cytoplasmic puncta indicated that 8.3 ± 3.6% colocalized with lysosomes. In cells transfected to express Cx43-GFP there was a higher percentage of colocalization of annular gap junctions with the LAMP1 lysosome marker than that found in cells expressing endogenous Cx43. We believe the higher percentage colocalization with Cx43-GFP was due to an increased aggregation of tagged Cx43 in the cytoplasm of cells overexpressing the Cx43-GFP and thus the possibility of false positives.

A pitfall of interpreting colocalization data is that at the light microscopic level structures that are separated by as much as 200 nm can still appear to be colocalized. Further, the capacity to distinguish and analyze the morphology of the organelles is limited by the resolution of images collected with light microscopy. Transmission electron microscopy was used to better resolve the morphology and to positively identify organelles and their associations.

The annular gap junction vesicle pentalaminar or dense double membrane was used to identify gap junction structures with TEM (Figure 1D–F and Figure 3B–G). Taking advantage of the morphological characteristic of the annular gap junction membrane and the well-known distinguishing characteristics of other cytoplasmic organelles, we thus analyzed the association of organelles with gap junction structures.

Annular gap junctional vesicles were observed to interact with lysosomes (Figure 3). Lysosomes were identified by their limiting membrane which is single, unlike that of annular gap junction vesicle and mitochondria, and the density of the material found within their lumen. Lysosome morphology however was variable and the density of the material within their lumen was dependent on the stages of digestion (Figure 3B–G). In annular gap junctional profiles that appeared to be undergoing degradation, the inner and outer annular gap junction membranes were separated from one another and the outer membrane was continuous with the lysosomal membrane (Figure 3B,D), as if the two distinct organelles were becoming one. Clathrin was found associated with the annular gap junction, even at the point that they were fused with lysosomes (Figure 3C,E). With ultrastructural analysis the number of lysosomal/annular vesicle associations could be quantitated in detail. Based on the analysis of 549 annular gap junction vesicles, we demonstrated that 2.6 ± 0.6% were fused with lysosomes.

#### 2.2.2. Mitochondria

In addition to colocalization with lysosomes, annular gap junctions were revealed with immunocytochemistry, super-resolution microscopy, and transmission electron microscopy (TEM) to colocalize with mitochondria (Figure 4). With immunocytochemistry the size of the puncta was used to identify a structure as an annular gap junction (Figure 4A). In Figure 4, colocalization of mitochondria detected with either antibody to Mitotracker (red) or TOM (red) and annular gap junctions detected with Cx43 antigen (green) was evident. As seen with super-resolution microscopy some mitochondria colocalize with more than one annular gap junction vesicle.

To visualize the fine structure morphology at the sites of physical association between the mitochondria and annular gap junctions, TEM imaging was used (Figure 4C and Figure 5). Mitochondria were observed to associate and in some cases to follow the contour of annular gap junction membranes (Figure 4C and Figure 5). Actual direct fusion of the outer membrane of the annular gap junction vesicles and mitochondria were sometimes evident (Figure 5). We selected a distance of less than 25 nm as indicative of contact area. This distance was selected based on the reported contact distance of 10–30 nm between mitochondria and surface gap junction plaques [43] as well as lysosomes [44,45]. The distances, all less than 25 nm, were averaged and the data is expressed as standard error from the mean. The average contact distance between mitochondria and annular gap junctions was 18.4 ± 0.7 nm with a range from 0–25 nm. The distances were calculated from four different TEM preparations with *N* = 200.

Ultrastructural analysis of 549 images of annular gap junctions revealed that 5.2 ± 1.1% associated with mitochondria. The finding that annular gap junction vesicles appeared to be in direct contact with mitochondria, was significantly greater than expected by random chance. The percentage of annular gap junction vesicles in contact with mitochondria (5.2 ± 1.1%) exceeded the percentage of annular gap junction vesicles that were found to associate with lysosomes (2.6 ± 0.6%).

To address the question of random vs. specific interaction of annular gap junctions and mitochondria, a Wilcoxon rank-sum nonparametric test was used to compare the mean number of annular gap junction/mitochondria contacts vs. annular gap junction vesicle/vacuoles contacts. Over a randomly selected 20 images, there were 188 total mitochondria and 240 total vacuoles. The number of mitochondria per image ranged from 0 to 39 with a mean of 9.4 ± 0.75, while the number of vacuoles ranged from 0 to 35 with a mean of 12 ± 0.65. In 35% of the images, there was at least one annular gap junction in contact with a mitochondrion while only one image indicated an annular gap junction contacting a vacuole. The mean number of annular gap junctions in contact with mitochondria was 0.346 ± 0.078 vs. 0.009 ± 0.007 in contact with a vacuole. A Wilcoxon rank-sum nonparametric test comparing the mean number of touches per image resulted in a statistically significant higher rate of annular gap junctions touching a mitochondria than a vacuole (*p* = 0.008), consistent with specific rather than random events.

To further address the concerns of random vs. nonrandom chance contact based on annular gap junction sizes, we measured annular gap junction profile sizes and compared these measurements to the average distance between mitochondrial membranes. If annular gap junction contact with mitochondria was random, then we would expect larger annular gap junctions to more frequently contact mitochondria than smaller ones. We found the average gap junction profile measured with TEM was 421 ± 16.9 nm (*n* = 199 annular gap junction). We arbitrarily selected 365nm, the median annular gap junction size, for classifying annular gap junctions as either large or small. Based on chi square analysis we saw no size-based difference (*p* = 0.83) in the frequency of finding a large (size 596.8 ± 21.7 nm) annular gap junction contacting a mitochondria (19, *n* = 100) than a smaller (size 244.2 ± 71.6 nm) annular gap junction contacting a mitochondria (19, *n* = 99). The average contact distance between mitochondria and the larger (18.7 ± 1.0 nm) or smaller (17.9 ± 0.9 nm) gap junctions was not significantly different, as calculated with a Student’s *t*-test (*p* = 0.57).

In images in which annular gap junctions were in the same field, the average minimal distance between the annular gap junction and the nucleus was 330.9 ± 96.7 nm. That the annular gap junction vesicles were not in contact with the relatively large nucleus would be consistent with the interaction of annular gap junctions with the mitochondria not being a random encounter based on the size of the annular but that a specific interactions between mitochondria and annular exist.

## 3. Discussion

In this study, we documented the presence of cytoplasmic Cx43-containing structures and we analyzed the details and frequency of interactions of mitochondria and lysosomes with annular gap junction vesicles. We found that the interactions between annular gap junctions and mitochondria were seen more frequently than between annular gap junctions and lysosomes. The behavior of the puncta believed to be annular gap junctions were analyzed with live cell imaging and it was determined that these structures could either (1) undergo fission [23], (2) fuse with other organelles, (3) disappear from view, or (4) appear to recycle back to the surface. In this study, to address the concern that in our live cell imaging studies GFP tag may either interfere with or alter organelle interactions with Cx43-containing structure, or that the Cx43 puncta were not positively identified as annular gap junction vesicles, we took advantage of TEM techniques to positively identify and analyze organelle association in cells expressing endogenous Cx43.

It was determined, based on ultrastructural analysis of the structures in the cytoplasm, that some of the puncta identified as annular gap junction at the light microscopic level of resolution could be cytoplasmic aggregates. It should be noted however that such structures were relatively rare, and thus our findings would be consistent with most of the puncta seen with live cell imaging being annular gap junctions.

It is well accepted that connexin in annular gap junction vesicles are degraded by lysosomal, proteasomal, or autophagosomal pathways [14,24,42,43,44,45]. Lysosomal degradation is based on TEM observations of colocalization of lysosomes and annular gap junction [8,14], the increase in annular gap junction vesicles when lysosomal activity is inhibited [44], and the demonstration of acid phosphatase activity in these annular gap junctions [8,14]. In this current study we did not analyze proteasomal or autophagosomal pathways but instead have characterized the most common degradation pathway, lysosomal degradation. We found with TEM, that only 2.6% of the annular gap junction vesicles analyzed were associated with lysosomes at a given point in time. Although this figure may suggest a limited contribution to the gap junction turnover or behavior, any alteration in the rate of Cx43 degradation might serve as a post-translational means of altering intercellular communication. While the association of lysosomes with annular gap junction appeared as expected to result in the fusion of the two organelles, the contact between mitochondria and annular gap junction vesicles did not result fusion to form one structure.

Although the close association of surface gap junction plaques and mitochondrial has been reported [43,46,47] the association of annular gap junction with mitochondria, to our knowledge, has not been previously characterized. Forbes and Sperelakis showed an annular structure that could be an annular gap junction that is associated with a mitochondrion [43]. However, this early study was before live cell imaging of tagged Cx43 was readily available and the authors speculated that the annular profile was an invaginated surface gap junction cut at an angle that made it appear to be annular. Although the association of mitochondria and annular gap junctions was not mentioned by Emdad and colleagues, an image demonstrating contact between these two organelles was seen in a TEM image in this paper [48]. The authors suggested the annular profiles could represent either sectioning through convoluted gap junction plaques or internalized annular gap junctions. In the current study we characterize the contact between annular gap junctions and mitochondria with TEM, immunocytochemistry, and super-resolution microscopy.

The contact between mitochondria and annular gap junctions viewed with TEM was a distance as close as 25 nm or less at the site of contact and in some cases the two membranes of the two organelles were actually in physical contact with one another. This contact distance is comparable to the reported distance of 10–30 nm measured between the mitochondria and gap junction plaques [43], lysosomes [44,45], or the endoplasmic reticulum (ER) [45]. The existence of ER–mitochondria contact sites has been well established by electron microscopy and time-lapse fluorescence microscopy and it has been suggested that in yeast, a network of contact sites serve to integrate the ER, vacuoles, and mitochondria [45]. Here we suggest that in addition to the interaction between ER and vacuoles, mitochondria also interact with annular gap junction vesicles. The benefit of this interaction can only be speculated at this point but it is tempting to suggest one possibility may be that Cx43 from annular gap junctions may be delivered to mitochondria when the two organelles come into physical contact. Future studies are needed to confirm this speculation. In addition to Cx43 from the annular gap junction being delivered to the mitochondrial membrane, it is also possible that connexins may arrive at the mitochondria in secretory vesicles from Golgi or ER interactions. We have not ruled out these possibilities.

It has been well documented however that there is Cx43 on mitochondrial membranes [49,50,51,52]. Proof of mitochondrial Cx43 have been provided from investigators who used a wide variety of different techniques, including immunocytochemical colocalization of Cx43 with mitochondrial proteins at the light microscopic and immunogold electron microscopic levels of resolution [49], flow cytometry studies, and quantitative Western blot analysis [49,50,51,52]. Mitochondrial Cx43 has been demonstrated to protect the heart from ischemic injury, facilitating mitochondrial movement to the cell periphery, as well as maintaining mitochondrial network integrity [49,50,53,54,55,56,57,58]. Further, mitochondrial metabolic activity and morphology alterations have been demonstrated in adipocytes from Cx43 knockout mice and knockdown cells in culture [54], consistent with there being a Cx43 mitochondrial functional dependency [49,56,57]. Additionally, since mitochondria are known to sequester calcium ions and to provide cell energy, it is possible that annular gap junction contact with mitochondria may function in regulating annular gap junction channels and/or ATP exchange.

Intervention studies that measure changes in mitochondria Cx43 relative to treatments that alter annular gap junction formation or degradation and thus increasing or decreasing annular gap junction number available to interact with the mitochondria are needed. Further, examining cell types that have different levels of gap junction plaque internalization would be useful. As to why some cell types internalize gap junctions more often than others is not clear, however there are a number of interventions that will alter this internalization process including treatments that modulate protein kinase expression [59,60]. For example, elevating Src and protein kinase C increases frequency of internalization [60], but elevating protein kinase A decreases internalization [61,62]. It is thought that this change in the frequency of internalization and thus annular gap junction formation is regulated by kinase mediated phosphorylation of the Cx43 proteins [61,62]. Treatments with kinase activators, inhibitors or the study of cells expressing the truncated or mutated Cx43 as well as those with autophagosomal markers or proteasomal markers are needed to increase our understanding of Cx43 phosphorylation and annular gap junction interactions with cytoplasmic organelles. Further, we have demonstrated that internalization involves clathrin and dynamin mediated mechanisms that if perturbed alters annular gap junction formation [23]. Zonula occludens-1 protein (ZO-1) has been demonstrated to bind to the C-terminal tail of Cx43 where it contributes to a number of Cx43 functions including, gap junction channel formation, docking and lateral movement to determine gap junction plaque size [9,63,64,65,66]. Cx43/ZO-1 binding is regulated via phosphorylation/de-phosphorylation of S373 by AKT kinase [67]. Src-mediated dissociation of ZO-1 initiates endocytic internalization of gap junctions and thus annular gap junction formation [64]. Gap junction plaque size has been demonstrated to be modulated by ZO-1 binding and it has been reported that tagging GFP to the C-terminal of Cx43 inhibits ZO-1 binding [64,68], increases gap junction plaque size and endocytosis resulting in increased size and number of annular gap junctions [64]. In contrast in the human heart, diminished ZO-1 levels coincide with reduced Cx43 staining [69]. The relationship between ZO-1 and gap junction size may vary depending on tissue types and pathological conditions. The SW-13 cells used in this study form large gap junctions [8]. While ZO-1 levels or the effect of expressing Cx43-GFP on annular organelle contacts or gap junction plaque size were not studied, at least for these cells that express only endogenous Cx43, the annular size does not appear to influence the number of mitochondria/annular gap junction contacts. In an early study, in which the occurrence of annular gap junction in various cell types was reviewed, it is apparent that some cell types have more annular gap junctions than other cell types [70]. While the SW-13 appear to form large gap junctions that are frequently internalized relative to many other cell types [8,70], there are no or few reports comparing annular gap junction formation in different cell types. The benefits of some cells having larger gap junctions are unknown. It is however presumed that these cells may be more dependent on cell–cell communication to maintain function. For example, gap junction plaques have been shown in vivo and in vitro to be larger in the ventricle than in atrial myocytes [69,71]. The function of large ventricular gap junctions has been speculated to play a critical role in intercellular current transfer, needed for normal ventricular function. Further, annular gap junction profiles increased when hypertrophy of the left ventricle was induced by abdominal aorta banding [48]. How the number of annular gap junctions in cells that have fewer or more annular gap junctions or that make larger or smaller surface gap junctions affects mitochondria function is unclear and can only be speculated. The factors regulating cell-specific frequency of internalization, annular gap junction vesicle size, and their subsequent interactions with mitochondria and lysosomes need to be investigated in a variety of cell types if we are to fully understand gap junction protein trafficking.

The method of Cx43 delivery to the mitochondria is not fully understood however it appears to involve heat shock protein 90 (Hsp90) and TOM 20 [39]. Further, it is known that mitochondrial Cx43 is phosphorylated [58], however it is not known which Cx43 amino acid residues are phosphorylated. The mechanism that regulates trafficking of Cx43 to the mitochondria and its protective role in pathophysiological conditions remain unknown. Is the annular gap junction vesicle possibly a cytoplasmic source of phosphorylated Cx43 that could potentially serve as a source for Cx43 distribution to other compartments? Although it is well accepted that connexins in annular gap junctions are degraded, other possible fates have received little attention despite the reports of Cx43 recycling from annular gap junctions to participate in rapid gap junction plaque [31,32,64,72].

The method by which Cx43 traffics to the mitochondria has not been elucidated here. However, the findings of Cx43 targeting to the mitochondria and Cx43-dependent changes in mitochondrial function and movement within the cytoplasm [49,50,53,54,55,56,57,58] points to the possibility that in addition to traditional Cx43 trafficking and function at the cell surface that Cx43 may traffic and function in noncanonical manners. Here we demonstrate the association of annular gap junctions with mitochondria and suggest that Cx43 in annular gap junctions may have a fate that is unrelated to degradation. Future studies are needed to elucidate the ‘tethering’ molecules that hold annular gap junctions to mitochondria and to clarify the need for the interaction annular gap junctions with mitochondria.

## 4. Materials and Methods

### 4.1. Cell Culture

SW-13 human adrenocortical tumor cells (American Type Culture Collection, Rockville, MD, USA) were cultured in L-15 medium which contained fetal calf serum (10%), penicillin (0.06 mg/mL), streptomycin (0.1 mg/mL), and Fungizone (0.01 mg/mL), buffered with l-arginine at pH 7.4 (reagents and medium from Invitrogen, Carlsbad, CA, USA). Cells were grown at 37 °C in a 5% CO_2_ atmosphere.

### 4.2. Gap Junction Antibodies and Probes

Affinity purified polyclonal rabbit antibodies (IgG) were prepared against synthetic peptides corresponding to the carboxyl terminus of the Cx43 molecule (residues 370 to 381) [73] (Zymed Laboratory, San Francisco, CA, USA). Preparation and characterization of these antibodies have been previously described [73].

### 4.3. Immunocytochemistry

Cells were prepared and stained with immunocytochemical techniques, as previously described [23] for connexin 43 (1:100; Proteintech, Rosemont, IL, USA), clathrin (Abcam, Cambridge, MA, USA), lysosomes with Lamp1 staining (Abcam), and mitochondrial proteins with Mitotracker (1:100) or Tom20 (Santa Cruz, Dallas, TX, USA) (1:1000). Prior to immunocytochemical procedures, the cells were grown on coverslips, rinsed with phosphate buffered saline solution (PBS), fixed at room temperature, in 3% formaldehyde for 20 min, permeabilized in cold acetone for 7 min, and then incubated at 37 °C for 1 h or at 4 °C overnight in the primary antibody. In some experiments colocalization studies where performed. In these procedures, labeling of connexin was followed by a 4 °C overnight incubation in clathrin antibody. In experiments to colocalize mitochondria, the cellular localization of Tom-20, or mitotracker was visualized with a rhodamine conjugated goat anti-mouse secondary antibody (1:1000; Pierce, Rockford, IL, USA). The coverslips were stained with Hoescht, washed in PBS, and placed onto glass slides with a drop of Fluoromount-G antiquench reagent (Southern Biotechnical Lab, Birmingham, AL, USA). Immunolabeled cells were imaged at 63X with an Olympus Provis microscope (Olympus, Center Valley, PA, USA) and with the MetaMorph Imaging System (Molecular Devices, Downington, PA, USA).

For immunocytochemical analysis, the number of gap junction plaques and annular vesicles per cell nuclei were calculated. Fluorescent spherical puncta that were greater-or-equal to 0.5 µm in diameter were classified as annular gap junction vesicles while plaques were identified by their typical elongated profile or by the presence of aggregated puncta (smaller plaques), that were obviously at the cell surface between two cell pairs. The data was expressed as the average number of annular gap junction vesicles per cell.

### 4.4. Percent Colocalization

The percent colocalization was calculated from 50 cells (10 cells were selected from each of five files) in images collected by confocal step through selecting elements at the mid-plane of the Z stack. All images were saved as tiffs prior to analysis. The MetaMorph Software Program was used to view and trace the cell borders and to determine the percent colocalization of cells positive for both signals. Specifically the merged image was separated into red and green channels and the trace areas were transferred to the separated images. After thresholding the images, the number of Cx43 containing objects was determined with the automatic counting tool in MetaMorph. The counts were displayed on the “record count screen”, which was then analyzed and the number of surface gap junctions (as well as the number of clusters or aggregates or any object that did not look like an annular gap junction) were counted manually and subtracted from the total number of objects. The number of overlapping areas (yellow objects) were then counted manually by analyzing the merged image. The number of annular gap junctions that colocalized with lysosomes or mitochondria were determined by counting the number of annular gap junctions that had any yellow associated with them. The data was expressed as the average percent colocalization of annular gap junction vesicles with lysosomes or mitochondria.

### 4.5. Transfection with cDNA

To visualize gap junction trafficking in living cells, adrenal cells were transfected with cDNA encoding the fluorescent Cx43-GFP (provided by Dr. M. Falk, Lehigh University, Bethlehem, PA, USA). The Cx43-GFP vector was constructed by linking the GFP fluorescent reporter protein to the C-terminus of the rat Cx43 cDNA, and it has been demonstrated to assemble into gap junction plaques similar to wild type Cx43 [23]. Lipofectamine^2000^ Transfection Reagent (Life Technologies, Grand Island, NY, USA) was used to establish cell populations that transiently expressed fluorescently tagged Cx43, GFP, or empty vector.

The day before transfection, cells were seeded onto coverslips or in Mattek cell culture dishes (MatTek Corporation, Ashland, MA, USA). Cell populations, at 70–80% confluence, were transfected in Opti-mem medium which contained Lipofectamine^2000^ Transfection Reagent (Invitrogen) and 4 µg of plasmid DNA (Cx43-GFP, or empty vector) for 4 h at 37 °C in an atmosphere of 5% CO_2_. The resulting complexes were removed by gentle aspiration and washed with PBS. Fresh L-15 complete cell growth medium was added to the dishes, and the cells were incubated at 37 °C in 5% CO_2_ for 24–48 h before imaging.

### 4.6. Imaging of Cx43-GFP in Living Cells

MatTek glass bottom dishes with cells expressing Cx43-GFP or empty vector were placed into a temperature controlled chamber and maintained at 37 °C in 5% CO_2_ on a live cell Nikon A1 series automatic confocal laser point scanning system (Nikon Instruments Inc., Melville, NY, USA). Image acquisition on the Olympus was performed with a MetaMorph Imaging System (Molecular Devices) and Nikon NIS-Elements on the A1 Nikon microscope.

Images obtained with a 63× oil objective with a numerical aperture of 1.4, were collected at 1 min intervals over a 26 h viewing period. DIC and fluorescent images were obtained using the standard fluorescence detector at wavelengths 482 and 595 for all experiments. Focus was maintained with the Perfect Focus System (PFS) function and images were acquired with Nikon’s NIS-Elements software. Qualitative and quantitative analyses were performed on the data sets with the rendering tool in NIS-Elements. Time-lapse images were used to analyze gap junction plaque internalization and for analysis. Time-lapse images were converted into movies and evaluated with the MetaMorph program (Molecular Devices).

Annular gap junction vesicle pattern of displacement within the cell and corresponding changes in size (area expressed as µm^2^) were quantitated with the tracking function in the Imaris analysis software (Bitplane Scientific, South Windsor, CT, USA). Selected annular vesicles were segmented based upon labeling intensity and then followed over time. Statistical significance of differences was determined with the Student’s *t*-test.

### 4.7. Super-Resolution Live Cell Microscopy

Super-resolution microscopy was used to analyze annular gap junction and mitochondrial interaction with the stimulated emission depletion (STED) microscopy technique. STED allows for enhanced spatial resolution of the mitochondria which improves our accuracy when analyzing annular gap junction associations with mitochondria across the *x*, *y*, and *z* planes. Gated STED images were obtained using a commercial Leica SP8 STED 3X system (Leica Microsystems, Mannheim, Germany) housing a 100× oil objective with a numerical aperture of 1.4. Connexin 43 and Tom-20 antibodies were used to label annular gap junctions and mitochondria, respectively, for STED imaging.

### 4.8. Transmission Electron Microscopy

Cell monolayers were briefly rinsed in PBS then fixed with 2.5% glutaraldehyde in PBS, pH 7.4, for 1 h at room temperature. All samples were then washed 3 times in PBS buffer and post-fixed for 1 h at 4 °C in 1% osmium tetroxide with 1% potassium ferricyanide. The samples were washed again and the cells were serially dehydrated in an ethanol (30%, 50%, 70%, and 90%) for 10 min followed by 15 min in 100% ethanol three times. The cells were then incubated in Epon 3 times for 1 h and finally embedded in resin to be sectioned. Ultrathin sections were cut, mounted on grids, and imaged on a JEOL 1011CX electron microscope (JEOL, Tokyo, Japan).

In Quantum dots protocols and in some protocols to view annular gap junction/organelle contacts, the cells were fixed (2% paraformaldehyde and 0.1% glutaraldehyde in PBS). Quantum dots (QDs) were used to specifically label connexin and clathrin. Adrenal cells were incubated in Cx43 or clathrin primary antibodies, followed by incubation in biotin IgG conjugated antibody, and then in a QD conjugated secondary antibody (Strepavidin conjugated with QD 655).

Two procedures were used to test the specificity of the quantum dot immuno-electron staining. First, the cells were incubated in the quantum dot-linked anti-rabbit immunoglobin without a preincubation of the thin sections in the first antibody. Second, the binding of quantum dot complexes to embedding resin was evaluated in areas free of cells.

### 4.9. Statistical Analysis

Data were analyzed with an unpaired two-tailed Student’s *t*-test, chi-squared test, or one-way analysis of variance (ANOVA). Wilcoxon rank-sum nonparametric test was used to compare the percentage of mitochondria/annular gap junction vesicles vs annular gap junction/vacuole contacts versus the percent expected by random chance. Data are presented as means ± SEM. In this analysis, out of 20 randomly selected images, there were 188 total mitochondria and 240 total vacuoles. The number of mitochondria per image ranged from 0 to 39 with mean = 9.4, while the number of vacuoles ranged from 0 to 35, mean = 12. The number of annular gap junctions was 0, 1, or 2. There were 10 images with at least one mitochondria and one annular gap junction and 11 images with at least one vacuole and annular gap junctions. These were used to compare annular gap junction contacts with mitochondria vs. vacuoles.

## Abbreviations

TEM	Transmission Electron Microscopy
QDs	Quantum Dots
AGJ	Annular Gap Junction Vesicle
